# Absence of neutrophils impairs the host defense in murine footpad model of chromoblastomycosis

**DOI:** 10.1371/journal.pntd.0012986

**Published:** 2025-04-23

**Authors:** Huan Huang, Minying Li, Yinghui Liu, Yangxia Chen, Zhenmou Xie, Mingfen Luo, Dongmei Li, Hongfang Liu, Liyan Xi

**Affiliations:** 1 Department of Dermatology, Dermatology Hospital of Southern Medical University, Guangzhou, China; 2 Department of Microbiology/Immunology, Georgetown University Medical Center, Washington, District of Columbia, United States of America; 3 Department of Dermatology, Sun Yat-Sen Memorial Hospital, Sun Yat-Sen University, Guangzhou, China; Universidad de Antioquia, COLOMBIA

## Abstract

Chromoblastomycosis (CBM), a chronic subcutaneous infection caused by black fungi such as *Fonsecaea monophora* (*F. monophora*), is characterized by a low cure rate, high recurrence rate, and prolonged treatment duration. Neutrophils, one of the most important innate immune cells, play complex roles in the prevention of fungal infections. This study investigated the function of neutrophils in host defense against *F. monophora* using a neutrophil-depleted mouse model and *in vitro* co-culture conditions. Fungal burden, histopathological changes, and cytokine profiles were compared between neutrophil-depleted mice and isotype control mice. Our findings demonstrated that neutrophil depletion in mice led to impaired fungal clearance, prolonged inflammation in *F. monophora* infected footpad tissues, highlighting the critical role of neutrophils in controlling *F. monophora* infection. Histopathological analysis revealed extensive inflammatory cell infiltration, especially macrophages, accompanied by elevated levels of pro-inflammatory cytokines such as IL-1β, CCL3, IL-6, and TNF-α. Besides, we observed that neutrophils play a key role in inhibiting the morphological transition of *F. monophora* from conidia to hyphae and sclerotic-like cells. Notably, the *F. monophora* morphology was also associated with the formation of neutrophil extracellular traps (NETs) in *in vitro* experiment. These findings underscore the importance of neutrophil-mediate immune responses in early fungal clearance and their ability to influence *F.monophora* morphological transition. The study provides novel insights into the immune mechanisms underlying CBM and highlights the potential therapeutic implications of targeting neutrophil-mediated responses in CBM infections.

## Author summary

Chromoblastomycosis (CBM) is a chronic subcutaneous infection caused by black fungi such as *Fonsecaea monophora* (*F. monophora*), known for its low cure rate, high recurrence, and prolonged treatment. Neutrophils, key players in innate immunity, play a crucial role in defending against fungal infections. This study investigated the function of neutrophils in host defense against *F. monophora* using a neutrophil-depleted mouse model and *in vitro* experiments. Results showed that neutrophil depletion impaired fungal clearance and prolonged inflammation in infected tissues, emphasizing their critical role in controlling *F. monophora* infection. Histopathological analysis revealed extensive inflammatory cell infiltration, particularly macrophages. Neutrophils also inhibited the fungal transition from conidia to hyphae and sclerotic-like cells. Notably, the *F. monophora* morphology was also associated with the formation of neutrophil extracellular traps (NETs) *in vitro* experiments. These findings reveal the importance of neutrophils in early fungal clearance and morphology regulation, offering insights into immune mechanisms and potential therapeutic strategies targeting neutrophil-mediated responses for CBM.

## 1 Introduction

Chromoblastomycosis (CBM) is considered as a neglected tropical disease (NTD) and refer to a chronic subcutaneous infection caused by several dematiaceous fungi [1,2], with the genus *Fonsecaea* being one of the common causative pathogens [3]*.* A recent re-analysis of ITS rRNA sequences from causative strains collected in southern China revealed that more than 80% of morphologically identified *F. pedrosoi* are actually *F. monophora* [4,5]. The low cure rate, high recurrence, and prolonged treatment course of this disease impose a heavy economic burden on patients and society in developing country [2]. From a clinical perspective, the progression of CBM characterized by a localized, diffuse lesion surrounding healthy skin in the early stages, followed by fibrous tissue proliferation in the central region of the lesion, ultimately resulting in scar formation in the chronic stage [2,6]. This clinical manifestation course may be attributed to the dynamic interplay between the pathogenic organisms and the host. A impaired fungal clearance by host immune cells underlies disease chronicity, highlights the significance of promote antifungal immune mechanisms, particularly in the early stage of the infection.

Several immune characteristics are believed to contribute to the chronicity of CBM, and the host immune mechanisms against CBM appear to be intricate [7]. Histopathology examination of the affected tissue area shows infiltration of a large number of mixed inflammatory cells, typically consisting of neutrophils (polymorphonuclear neutrophils, PMNs), mononuclear phagocytic cells, and lymphocytes [8]. Interestingly, macrophages were reported to play a fungal-static role rather than a fungicidal role in CBM [9], and are more involved in tissue fibrosis [10]. The adaptive immune response is predominantly characterized by a Th2 cell response, which is less effective at eliminating pathogens compared to Th1 cell response [10,11]. While the roles of immune cells such as macrophages and T lymphocytes are well studied, the role of neutrophils in CBM has been less explored, and their function and mechanisms in contributing to the chronicity of CBM remain unclear.

Neutrophils act as the first line of defense in innate immunity and contribute to fungal elimination through mechanisms such as phagocytosis, the release of reactive oxygen species (ROS), degranulation, and the formation of neutrophil extracellular traps (NETs) [12]. The NETs can entrap and kill fungi and bacteria without direct phagocytic uptake [13–15]. Neutropenia is therefore a well-established risk factor for invasive fungal infections, including candidiasis, aspergillosis, and mucormycosis [16,17]. Neutrophils are not only crucial in invasive fungal infections but also play positive roles in subcutaneous fungal infections, such as *Sporothrix schenckii* infection in both humans and animals [18,19]. While in an *in vitro* study, phagocytosed *S. schenckii* spores were observed to still germinate within neutrophils, suggesting that neutrophils exhibit relatively weak fungicidal activity in this context [20].

In our recent study on CBM infection, we found substantial neutrophil infiltration and NETs formation in 90.0% of CBM granuloma cases [10]. Other studies have reported that sclerotic cells and their cell walls appear to be continuously targeted by neutrophils in CBM [21], and neutrophilic infiltration was responsible to prevent *F. pedrosoi* spreading [22]. These *in vivo* data align with findings from *in vitro* experiments showing that neutrophils have some capacity to phagocytose and exert oxidative killing effects on *Fonsecaea* spp. Conversely, in the mouse model of *F. pedrosoi* infection, neutrophils were reported to favor the spread of fungal cells and contribute to the chronicity of the infection [23]. In extreme cases of neutrophil over-infiltration and excessive ROS production, abundant neutrophil infiltration can even lead to tissue necrosis [24]. For instance, depletion of neutrophils with anti-Ly6G antibody attenuated lung damage in an acute lung injury model induced by intranasal flagellar hook protein E [25], and excessive ROS release from neutrophils in pulmonary tissue contributed to tissue damage in a mouse *Acinetobacter baumannii* pneumonia model [26].

These variable, or even contradictory neutrophil responses to different pathogens warrant the further characterization of the precise role of neutrophils in the infection process of CBM. Specifically, it remains unclear whether neutrophils primarily contribute to fungal clearance or, conversely, facilitate fungal dissemination. To elucidate the role of neutrophils, this study established a footpad *F. monophora* infection model in neutrophil-depleted mice to investigate the function and mechanisms of neutrophils in host clearance of *F. monophora.* Additionally, through *in vitro* co-culture of *F. monophora* with neutrophils isolated from healthy donor peripheral blood, the relationship between neutrophil NETs formation and *F. monophora* morphology was explored.

## 2 Materials and methods

### Ethics statement

This study was conducted in strict accordance with the ethical guidelines and regulations for animal research, adhering to the principles of the “3Rs” (Replacement, Reduction, and Refinement). All animal experiments were conducted in accordance with the National Institutes of Health guidelines. All experimental protocols involving animals were reviewed and approved by the Ethics committee of the Dermatology Hospital, Southern Medical University with approval number 2018002 (ethics amendment dated 7 March 2018). Efforts were made to minimize animal suffering, including the use of appropriate anesthesia, analgesia, and humane endpoints. Experiments complied with international standards and China’s regulations on experimental animals (8/1/2011 C-WISC).

### 2.1 Fungal strain and growth conditions

#### 2.1.1 Preparation of *F. monophora* conidia and short hyphae.

*F. monophora* wild strain (CBS 269.37) used in this study was isolated in South America in 1936, and characterized by de Hoog *et al.*[27]. The strain was cultivated on potato dextrose agar (Becton, Dickinson and Company, USA) at 26°C for 10 days. Conidia were collected by flooding the culture surface with sterile PBS and filtered through a 20-μm filter. The fungal suspension was centrifuged at 12,000 rpm for 10 min at room temperature and pellet was resuspended in PBS. Conidia concentration was determined using a hemacytometer and adjusted to 2×10^7^/mL. To obtain hyphal fragments, the conidia were cultured in Sabouraud broth (SDB) for 14 days at 28°C with shaking. The resulting hyphal culture was sequentially filtered through 40 μm and 20 μm cell strainers to obtain a hyphal inoculum ranging in size from 20 μm to 40 μm.

#### 2.1.2 *In vitro* induction of sclerotic cells.

To induce sclerotic cells, the 14-days-old *F. monophora* hyphal fragments grown in SDB were collected using a glass homogenizer and adjusted to a final concentration of 1×10^5^/mL. Then, 100 μL of hyphal fragments was re-inoculated into 30 mL of synthetic basal medium (ATCC medium 830) at pH 5.5, with composition (g/L) as follows: 0.1g MgSO_4_, 1.5g NH_4_NO_3_, 1.8g KH_2_PO_4_, 5×10^-5^g biotin, 1.0×10^-4^g thiamine-HCl, and 6.5g glycerol, as previously described [28]. Additionally, Nikkomycin Z (MedChemExpress, #HY-19593) was added at a final concentration of 50 μg/mL. After a 60-days incubation period at 35.5°C, the sclerotic microcolonies were gently ground using a glass grinder to disperse the cells. The resulting cell suspension was filtered through a 20 μm membrane filter to obtain a suspension of individual sclerotic cell.

### 2.2 Animal model

#### 2.2.1 BALB/c mice infected by *F. monophora.*

Specific pathogen-free (SPF) female BALB/c mice (6-weeks old, weighing 19-21 g) were obtained from the Animal Center of Southern Medical University. The mice were randomly assigned into three groups: control group, isotype group, and neutrophil depletion group (15 mice per group). Footpad infection of isotype group and neutrophil depletion group was achieved by injecting 50 μL of the fungal solution containing 2×10^7^ *F. monophora* conidia into the plantar cushion of the mice. The control group received an equivalent volume of PBS injection.

To assess footpad inflammation, the thickness of the footpad was measured every other day using caliper. At 3, 7, 14, 21 and 28 days post injection (dpi), the infected mice were euthanized to collect footpad samples for subsequent analyses, including colony-forming unit (CFU) counting, histopathological examination, and cytokine measurement.

#### 2.2.2 Neutrophil depletion.

Two antibody clones, RB6-8C5 (anti-Grl) and 1A8 (anti-Ly6G), were used for neutrophil depletion as described in previous studies [29,30]. The choice of clone 1A8 in this study is based on its specificity for neutrophils. Studies have shown that this clone of anti-Ly6G is more effective at depleting neutrophils when compared to anti-Grl in [30,31]. To achieve sustained and controlled depletion of neutrophils in mice, a high-dose regimen of 1A8 antibody was employed in this study.

Mice received an intraperitoneal (i.p.) injection of 100 μg monoclonal anti-mouse Ly6G (1A8 clone; Biolegend, #127650) 24 hours before fungal infection. To maintain durable and effective neutrophils depletion, subsequent 25 μg or 50 μg of monoclonal anti-mouse Ly6G were intraperitoneally administered every other day in neutrophils depletion group (S1 Fig). The isotype group received an equivalent amount of isotype control antibody and the PBS group received an equal volume of PBS buffer. To confirm neutrophil depletion, peripheral blood was collected from mice by tail vein sampling at 0, 3, 7 and 21 dpi. Neutrophil levels in the blood were assessed by flow cytometry (BD FACS Celesta) using anti-CD45-FITC monoclonal antibody (eBioscience, #12-0451-81), anti-CD11b-Alexa Fluor 647 monoclonal antibody (eBioscience, #17-0112-81) and anti-ly6G-PE monoclonal antibody (eBioscience, #11-9668-80). Data were analyzed using FlowJo V10 software.

#### 2.2.3 Fungal burden assessment.

The fungal burden was measured by CFU counting. After euthanizing the animals, the footpads were excised, weighed, and homogenized in 1 mL of PBS. Following serial dilutions, homogenates were spread onto potato dextrose agar (PDA) plates supplemented with 0.2 M cefotaxime sodium and incubated for 7 days at 26°C.

#### 2.2.4 Histopathological staining.

For histopathological examination, footpad tissues were fixed in 10% formalin and embedded in paraffin and sectioned. The sections were stained with hematoxylin-eosin staining (H&E) or periodic acid-Schiff (PAS). All the sections were digitally scanned using a Hamamatsu Nano Zoomer (HAMAMATSU, Japan) for further analysis.

#### 2.2.5 Cytokine analysis.

The footpad samples were preserved in PBS containing a protease inhibitor cocktail. After homogenization, the cell suspension was centrifuged (4˚C, 16,000g, 10min), and the resulting supernatant from each sample was analyzed for neutrophil-related chemokines (GM-CSF, CXCL1, CXCL2, and CCL3), and inflammatory cytokines(IL-17A, TNF-α, IL-1 β, IL-6, and IL-10). The Cytokine & Chemokine 26-Plex Mouse ProcartaPlex (Invitrogen, #EPX260-26088-901) was used to quantify the cytokines according to the manufacturer’s instructions.

### 2.3 NETs *in vitro* experiments

#### 2.3.1 Isolation of neutrophils from peripheral blood of healthy individuals.

Human neutrophils were isolated using PolymorphoPrep density gradient media (#1114683; PROGEN Biotechnik GmbH, Heidelberg, Germany) immediately after peripheral blood collection, following the manufacturer’s protocol. Briefly, 5mL of anticoagulated whole blood was layered on top of an equal volume PolymorphoPrepin a centrifuge tube and centrifuged at 500g for 35 min at room temperature. After centrifugation, two distinct leukocyte bands were visible: the upper band at the sample interface containing mononuclear cells and the lower band containing neutrophils. The lower neutrophils band was collected and centrifuged at 200g for 5 min. The purity of the neutrophil preparation was determined using the Swiss Giemsa staining method or by flow cytometry with the following antibodies: anti-Hu-CD45-FITC (Biolegend, #368507), anti-CD11b-Alexa Fluor 647 (Biolegend, #101220), and anti-CD66b-PE (Biolegend, #392903).

#### 2.3.2 NETs visualization.

Neutrophils (2×10^5^ cells/24-well plate) were treated with *F. monophora* conidia (MOI=10), hyphae fragments (MOI=1) or sclerotic cells (MOI=1) for 4 hours on poly-L-lysine-treated coverslips. Additionally, neutrophils were treated with PMA (100 nM) or *C. albicans* (MOI=1) as a positive control and unstimulated neutrophils served as a negative control for NETosis. For confocal imaging, samples were fixed with 4% paraformaldehyde and then and stained with Calcofluor white (CFW) for 10 minutes at room temperature. After three washes, DNA was stained with Cytox Green nucleic acid stain (Invitrogen S7020) for 15 minutes. Samples were visualized suing a confocal microscope (Nikon, Tokyo, Japan; Model A1R). For Scanning Electron Microscopy (SEM) imaging, samples were fixed in 2.5% glutaraldehyde. Grids for electron microscopy were prepared at the Electron Microscope Core Facility of Southern medical University, and imaging was performed using a Hitachi S-3000 transmission electron microscope.

#### 2.3.3 NETs quantification.

Quantification of NETs was performed using PicoGreen to detect free double-stranded DNA (dsDNA), following the manufacturer’s protocol. Neutrophils (2×10^4^ cells/96-well plate) were co-cultured with *F. monophora* conidia (2×10^5^), or hyphae (2×10^4^) fragments or sclerotic cells (2×10^4^)for 4 hours. Neutrophils were treated with PMA (100nM) or *C. albicans* (2x10^4^) for positive controls of NETosis, while unstimulated neutrophils were used as negative control. The supernatant was gently removed and replaced with 100 µL of TE buffer. An equivalent amount of 1X Quant-iT PicoGreen reagent was added to the 96-well plates, followed by incubation for 5 min at room temperature. Free dsDNA was measured by fluorescence (480/520 nm) using a microplate reader (BioRad, USA).

### 2.4 Statistical analysis

All data are expressed as the mean ± SEM. Unpaired Student’s t-test with two-tailed P-values and one-way ANOVA test were used for statistical analyses unless indicated otherwise (GraphPad Prism 8.0 software). In all tests, P-values less than 0.05 were considered statistically significant.

## 3 Results

### 1A8 antibody treatment induced severe neutropenia in mice

To investigate the crucial roles of neutrophils in fungal clearance, mice were intraperitoneally injected daily with the neutrophil-specific antibody 1A8 to deplete neutrophils. Peripheral blood samples were collected from the tail vein at 0, 3, 7and 21 days post the first infection of *F. monophora* and analyzed using flow cytometry. The percentage of neutrophils (CD11b^+^Ly6G^+^) gated from CD45^+^ leukocytes (S2 Fig) decreased to 0.04% of 1A8 treatment, suggesting that we have successfully created a neutrophil depletion condition for the following experiments.

### Neutrophil depletion impairs *F. monophora* clearance in the footpad infected mouse model

**Fig 1:**
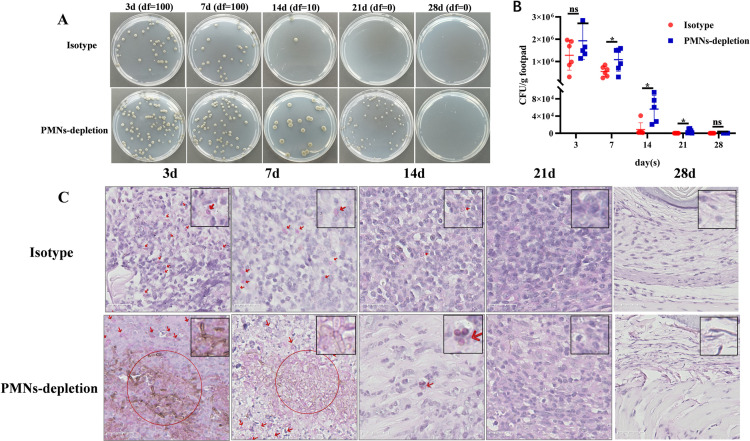
Neutrophil depletion impairs *F. monophora* clearance in footpads of infected mice. (A) Representative images of *F. monophora* colonies grown from footpad tissue homogenates on PDA for 10 days. Df: dilution factor. (B) The fungal burden of footpads presented by CFU was significantly increased in the PMNs-depletion group when compared with the isotype group. All comparisons were performed using two-tailed t-test (*P<0.05). (C) PAS staining of the footpads to showing different morphological features of *F. monophora*. The small arrow indicates single conidia or short hyphae; the circle highlights clumps of short hyphae; and the large arrow points to the sclerotic-like cells. The scale bar of the master figure is 25 μm.

The CFU values of *F. monophora* in footpads from the isotype group and the PMNs-depletion groups were calculated at 3, 7, 14, 21 and 28 dpi. The CFU values in the isotype group were significantly lower than those in the PMNs-depletion group at 7, 14 and 21 dpi (**Fig 1A and 1B**). Notably, at 14 and 21 dpi, almost no colonies were observed in the isotype group, whereas a large number of colonies formed in PMNs-depletion group (**Fig 1A**).

PAS staining of footpad sections revealed that the quantity of *F. monophora* followed a trend similar to the CFU values. Particularly at 3 and 7 dpi, a higher abundance of *F. monophora* spores and hyphae was detected in footpads from the PMNs-depletion group (**Fig 1C**). Compared to the isotype group, which primarily exhibited conidia, clusters of short hyphae and sclerotic-like structures were observed in the PMNs-depletion group. These findings underscore the essential role of neutrophils in controlling and limiting *F. monophora* germination during infection (**Fig 1C**).

### Neutrophil depletion exacerbates and prolongs the inflammatory response to *F. monophora*

**Fig 2:**
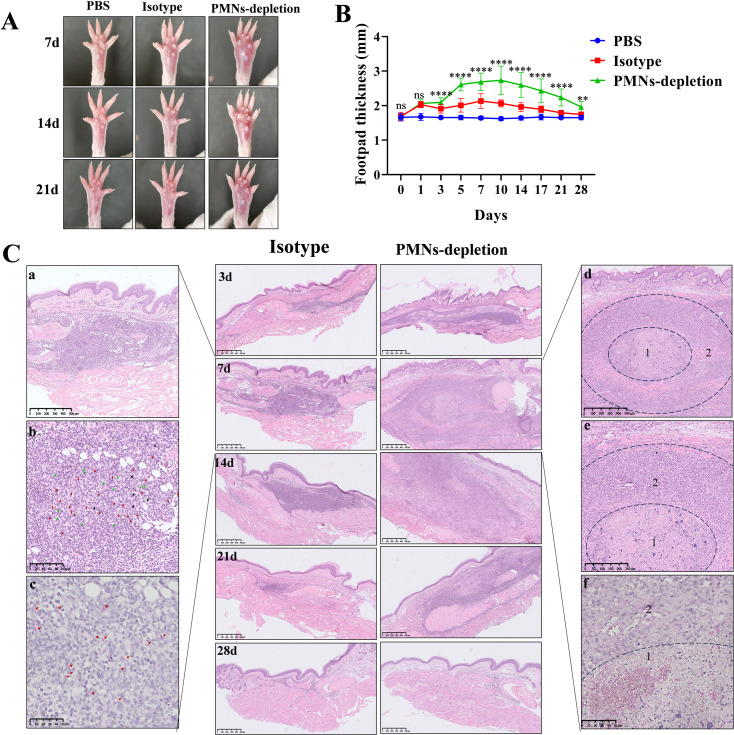
Impact of neutrophil depletion on inflammatory cell infiltration in the *F. monophora* infected mouse footpads. (A, B) The footpad thickness of mice was photographed and measured. The footpad thickness of mice in the PMNs-depletion group was significantly greater than that in the isotype group. All comparisons were performed using two-tailed t-test (PMNs-depletion vs. isotype group; *P<0.05; **P<0.01; ***P<0.001). (C) Middle panels: Histological features of inflammatory cell infiltration observed through HE staining at 3, 7,14,21 and 28 dpi. The scale bar is 500 μm. Left panels: Representative inflammatory cell infiltration in the footpad after *F. monophora* infection at 7 dpi. a & b: Massive mixed inflammatory cell infiltration in isotype group. Red arrows: neutrophils; green arrows: macrophages; black arrows: lymphocytes. c: PAS staining of *F. monophora* conidia. Red arrows: conidia. Right panels: Representative inflammatory cell infiltration in the footpad after neutrophil depletion at 7 dpi. d & e: 1- zone of degenerating neutrophils,2 - region of macrophages. f: The center of the inflammatory infiltration where large numbers of conidia and hyphae were observed by PAS staining. The scale bar for panels a&d are 500 μm, b&e are 250 μm, and c&f are 50 μm.

Both the PMNs-depletion and isotype groups exhibited inflammatory responses in *F. monophora*-infected footpads, characterized by visible tender swelling (**Fig 2A**). However, the infected course and severity of infected differed between the two groups. In isotype group, footpads swelling began at 1 dpi, peaked at7 dpi and gradually subsided by14 dpi. In contrast, the PMNs-depletion group also showed swellingat1 dpi, but it peakedat10 dpi and persisted until 21 dpi. Moreover, the degree of swelling in the PMNs-depletion group was significantly more pronounced than in the isotype group from3 dpi to 28 dpi. (**Fig 2B**).

Histopathological analysis revealed more extensive and persistent inflammatory cell infiltration in footpads from the PMNs-depletion group to the isotype group (**Fig 2C**). In the isotype group, abundant neutrophils infiltration, mixed with moderate macrophages and lymphocytes (Fig 2C**-2a and** 2C**-2b**),was observed alongside scattered *F. monophora* conidia at the peak of 7 dpi (Fig 2C**-2c**). In contrast, footpads from the PMNs-depletion group displayed a considerable number of neutrophil debris at the center of inflammatory cell infiltration (Fig 2C**-2d and** 2C**-2e**), entangled with masses of *F. monophora* conidia and hyphae mass (Fig 2C**-2f**), and surrounded exclusively by macrophages with minimal lymphocytes infiltration. These findings further confirmed that neutrophils depletion in tissues results in an incomplete immune response, impairing effective *F. monophora* clearance and exacerbating the duration and severity of infection.

### Cytokine profile analysis

**Fig 3:**
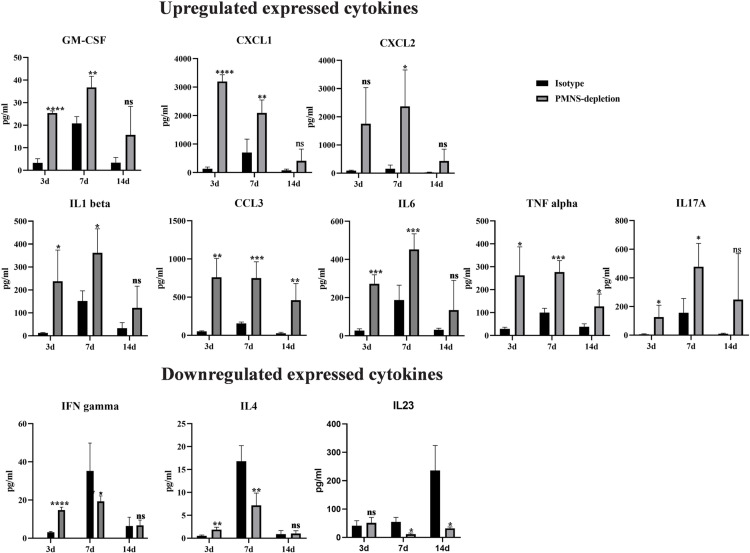
Cytokine expression profiles in the mouse footpads under PMNs-depletion condition compared to the isotype group. Upregulated expressed cytokines (GM-CSF, CXCL1, CXCL2, IL-1β, CCL3, IL6, TNF-α and IL17A) and downregulated expressed cytokines (IFN-γ, IL4 and IL23) were observed in PMNs-depletion group. All comparisons were performed using a two-tailed t-test (PMNs-depletion vs. isotype group; *P<0.05; **P<0.01; ***P<0.001). n=3.

Consistent with the abundant neutrophil debris observed in HE-stained footpads from PMNs-depletion mice (**Fig 2C**), the cytokine profile indicated a robust neutrophil chemotaxis response, characterized by elevated levels of GM-CSF, CXCL1 and CXCL2 from 3to 7 dpi. These cytokines likely represent a compensatory response following neutrophil destruction. Besides, in line with the observed increase in macrophages and monocytes, cytokines such as IL-1β,IL-6, CCL3, and TNF-α were highly expressed in the PMNs-depletion group (**Fig 3**).

Levels of proinflammatory cytokines associated with Th1/Th2 or Th17 responses, including IFN-γ, IL-4 and IL-23, were found to be lower in the PMNs-depletion group compared to the isotype group. However, expressions ofIL-17 were elevated in the PMNs-depletion group (**Fig 3**). These cytokine changes align with the paucity of lymphocytes observed in the PMNs-depletion footpads by HE staining.

### NETs induced by *F. monophora* conidia *in vivo* and induced by short hyphae and sclerotic cells *in vitro*

**Fig 4:**
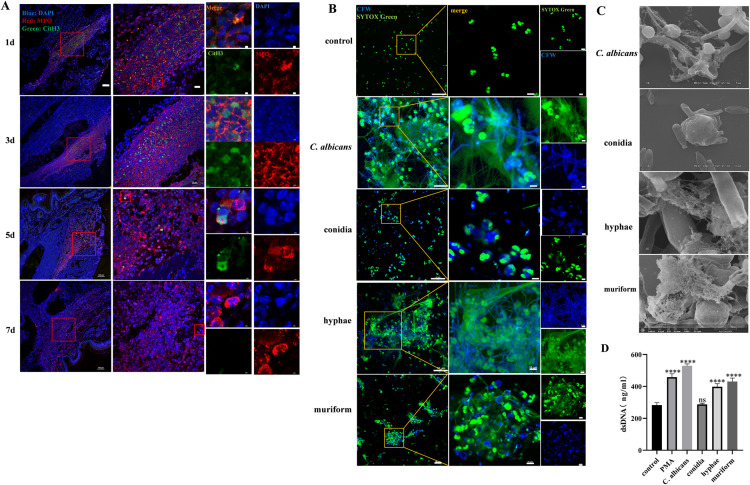
NETs induced by *F. monophora* conidia *in vivo* and by short hyphae and sclerotic cells *in vitro.* (A) NETs formation in mouse footpads was observed by confocal fluorescence staining at 1,3,5 and 7 dpi of *F. monophora* infection. Co-localization of citrulline histones (CitH3, green) and myeloperoxidase (MPO, red) marked NETs formation. DNA was stained with DAPI (blue). Scale bars, 100 μm (left), 20 μm (middle) and 2 μm (right). (B) Fluorescence staining was used to visualize NETs formation induced by *F. monophora* conidia, hyphae and sclerotic cells *in vitro*. Green: Sytox Greeen (DNA marker), blue: Calcofluor white (CFW, marking fungi). Scale bars, 50 μm (left), 10 μm (middle and right). (C) Scanning electron microscope (SEM) was used to observe NETs generation; (D) NETs were quantified by DNA concentrations in culture supernatants. All comparisons were performed using two-tailed t-test (vs. control group; *P<0.05; **P<0.01; ***P<0.001, ****, *P*<0.0001). Neutrophils treated with PMA or *C. albicans* served as positive controls, while unstimulated neutrophils were used as a negative control for NETosis.

Using immunofluorescence staining, NETs formation was assessed in *F. monophora*-infected footpads of mice in isotype group at 1, 3, 5 dpi. NETs were identified by the co-localization of citrullinated histones (CitH3) and myeloperoxidase (MPO) (**Fig 4A**). NETs were most prominent at 3 dpi but no longer detectable at 7 dpi.

After a 60-days induction period *in vitro,* morphological changes in *F. monophora* sclerotic cells were observed in ATCC medium 830 supplemented with Nikkomycin Z (S3 Fig). To further explore the impact of different *F. monophora* morphologies on NETs production, in an *in vitro* co-culture model, peripheral blood neutrophils from healthy donors were co-cultured with *F. monophora* conidia, hyphae and sclerotic cells for 4 hours to assess NETs formation.

Surprisingly, no NET-like structures were observed under confocal microscopy (**Fig 4B**) or scanning electron microscopy (**Fig 4C**) when challenged peripheral blood neutrophils with *F. monophora* conidia. However, NET-like structures were clearly evident when those neutrophils were stimulated with *F. monophora* short hyphae and sclerotic cells. Moreover, neutrophils stimulated with short hyphae and sclerotic cells released significant amounts of extracellular DNA, a hallmark of NETs. In contrast, the extracellular DNA released by neutrophils co-cultured with conidia showed no statistically different compared to the negative control group (**Fig 4D**).

## 4 Discussion

Neutrophils act as the first line of defense in innate immunity, playing a multifaceted role in combating pathogens. While neutrophils primarily function in pro-inflammatory and antimicrobial capacities, a set of neutrophils exhibit anti-inflammatory properties, contributing to inflammation resolution and tissue repair [12,32]. In extreme cases, neutrophil infiltration can even lead to tissue necrosis [24]. To elucidate the role of neutrophils in CBM, this study established a footpad *F. monophora* infection model in neutrophil-depleted mice to investigate the function and mechanisms of neutrophils in host clearance of *F. monophora*. Furthermore, through *in vitro* co-culture of *F. monophora* with peripheral blood neutrophils, the interplay between NETs formation and *F. monophora* morphology was explored.

Our findings demonstrated a significant impairment in the clearance of *F. monophora* from the footpads of neutropenic mice, accompanied by a prolonged disease course, thereby underscoring the pivotal role of neutrophils in controlling fungal infections. These findings provide *in vivo* experimental validate the *in vitro* findings in our earlier studies, confirming the role of neutrophils in CBM as antifungal effector cells [33,34]. Furthermore, our study reveals that neutrophils play a crucial role in reducing tissue swelling and mitigating the extent of inflammatory infiltration. Interestingly, Breda LCD *et al.* research suggested that neutrophils impaired the host’s ability to clear *F. pedrosoi* [23], which is in contrast to our findings that neutrophils continued to exhibit antifungal activity. This discrepancy may be attributed to the use of distinct mouse infection models, or variations in the fungal strains employed.

The NETs mechanism has been well studied in capturing, killing and degrading pathogens by constraining and inhibiting their proliferation [35,36]. Despite our earlier study reporting NETs formation in approximately 90.0% of CBM granuloma cases [10], only a small number of NETs were observed during the early stages of *F. monophora* conidia infection in the mouse footpad model in this study. While *in vitro* experiments demonstrated that NETs formation was induced exclusively when neutrophils were challenged with *F. monophora* short hyphae and sclerotic cells, but not conidia, highlighting the morphological dependence of NETs induction by *F. monophora*. This finding aligns with previous studies, including one by Breda *et al*., which reported a similar morphological dependency in *F. pedrosoi* NETs formation [33]. Furthermore, Branzk *et al*. suggested that neutrophils can selectively release NETs in response to pathogens with larger dimensions [37]. The inability of conidia to trigger NETs formation may represent an immune evasion strategy employed by *F. monophora* conidia, a hypothesis that requires further investigation. The discrepancy between the limited NETs formation observed *in vivo* during early infection and the absence of NETs formation *in vitro* could be attributed to differences between *in vivo* and *in vitro* environments,

Morphological transition represents a critical adaptive mechanism during host invasion by fungal pathogens process [38]. The reproduction and germination of conidia are fundamental to survival and pathogenicity of fungal pathogens in both environmental and host contexts [39]. Surprisingly, our findings reveal that neutrophils can effectively inhibit the morphological transition of *F. monophora* conidia into hyphae and sclerotic-like cells. Notably, when embedded in host tissue, most etiological agents of CBM including *F. pedrosoi,* undergo a transformation into the parasitic form, specifically the sclerotic cell form [2,40,41]. In contrast, hyphae and conidia are rarely observed in lesions [42], suggesting that this morphological shift enhances the ability to evade host immune defenses, contributing to the chronic and refractory of CBM [2,40,43]. Sclerotic cells, also known as muriform cells, are highly resistant to host immune responses and may exhibit reduced susceptibility to antifungal treatment [2]. Currently, there still a limited number of studies on the neutrophils inhibition of fungal germination, such as in *A. fumigatus* [44], but little is known about this process in CBM pathogens. This study provides direct evidence of sclerotic body germination in footpads infected with *F. monophora* following neutrophil depletion. These findings highlight the need for further investigation the morphological transition and immune evasion mechanisms of CBM.

In neutropenic mice, inflammatory response to *F. monophora* appeared more pronounced, as evidenced by increased levels of pro-inflammatory cytokines and chemokines, along with a significant influx of inflammatory cells. Notably, *F. monophora* in neutrophil debris were surrounded by a large number of macrophages. Correlating to the increase in macrophages, elevated levels of macrophage-associated cytokines, including GM-CSF, IL-1β, CCL3, IL-6 and TNF-α were observed in neutropenic mice. These cytokines are known to play a critical role in fungal clearance [45–48], and their upregulation stimulated a robust immune response.

In contrast to the prominent role of macrophages, the obvious reduced number of lymphocytes in neutropenic mice was associated with decreased expression of IFN-γ, IL-4 and IL-23, suggesting that the function of the adaptive immune system is compromised following neutropenia. Previous studies have also shown that under certain specific conditions, neutrophils can promote the proliferation of lymphocytes [49,50]. Based on the above findings, it is inferred that macrophages, rather than lymphocytes, may play the primary role in the eventual clearance of *F. monophora* in neutropenic mice. However, the antifungal activity of macrophages in CBM seems inefficient [9], as evidenced by the fact that despite a significant recruitment of macrophages to the infected tissue by 7 dpi, complete clearance of *F. monophora* was not achieved until 28 dpi. This further underscores the critical role of neutrophils in the early-stage clearance of *F. monophora*. The significantly elevated levels of GM-CSF, CXCL1, and CXCL2—key chemotactic factors associated with neutrophil recruitment—are likely a compensatory response to neutrophil depletion in the tissue. Although a significant reduction in lymphocyte counts was observed, an unexpected slight increase in IL-17A levels was noted. While previous studies have shown that IL-17A plays a role in neutrophil recruitment [50], its elevation in this context may also reflect a compensatory mechanism triggered by neutrophil depletion.

Furthermore, we acknowledge certain limitations in this study. Although the neutrophil depletion model is useful, it may not fully capture the complexity of neutrophil dynamics in human infections, as the model relies on antibody-mediated depletion, which could lead to off-target effects or incomplete depletion [30]. In addition, the murine model cannot entirely replicate the chronic characteristics and tissue-specific responses observed in human CBM [22]. Therefore, further research is needed to refine these models to enhance our understanding of disease mechanisms in humans.

In conclusion, we successfully established a neutrophils-depletion *F. monophora* footpad infection model. Using a neutrophil-specific anti-Ly6G antibody, we demonstrated that neutrophils play a crucial role in *F. monophora* clearance during the early stages of inflammation by shortening the disease course and reducing the severity of the inflammatory response. Moreover, neutrophils were found to play a critical role in suppressing the morphological transition of *F. monophora* from conidia to hyphae and sclerotic-like cells *in vivo*. These findings highlight the critical role of neutrophil-mediated immune mechanisms in CBM and suggest potential therapeutic implications for targeting neutrophil responses in CBM infections.

## Supporting information

S1 Fig 1A8 antibody treatment schematic diagram. (TIF)

S2 Fig Mouse peripheral blood neutrophil depletion model was successfully constructed. (**A**) Flow chart of representative neutrophils (CD11b^+^Ly6G^+^) as a percentage of leukocytes (CD45^+^) in mice receiving PBS, isotype control and 1A8 antibody (PMNs-depletion) for 3 days. (**B**) Statistical histogram of neutrophil percentages in peripheral blood of mice under different treatments. Statistical significance was determined by one-way ANOVA test (*, *P*<0.05; **, *P*<0.01; ***, *P*<0.0001, ****, *P*<0.0001.)
(TIF)

S3 Fig *In vitro* induction of sclerotic cell formation. *F. monophora* conidia or short hyphae were successfully transformed into sclerotic cells after 60 days induction *in vitro*.
(TIF)

S1 Data Raw data of the images involved in this study. (XLXS)
