# Supplementary figures and images for: Absence of neutrophils impairs the host defense in murine footpad model of chromoblastomycosis
Source: PLoS Negl Trop Dis. 2025 Apr 23;19(4):e0012986. doi: 10.1371/journal.pntd.0012986 (PMC12017585; doi:10.1371/journal.pntd.0012986)

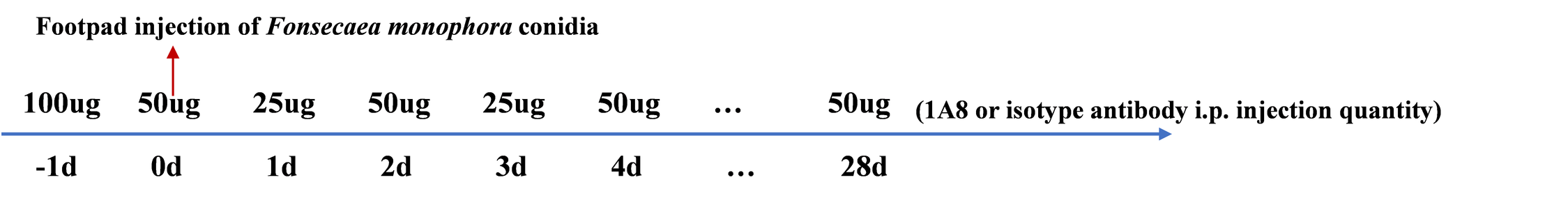

Supplement: S1 Fig — (TIF) [file pntd.0012986.s001.tif]

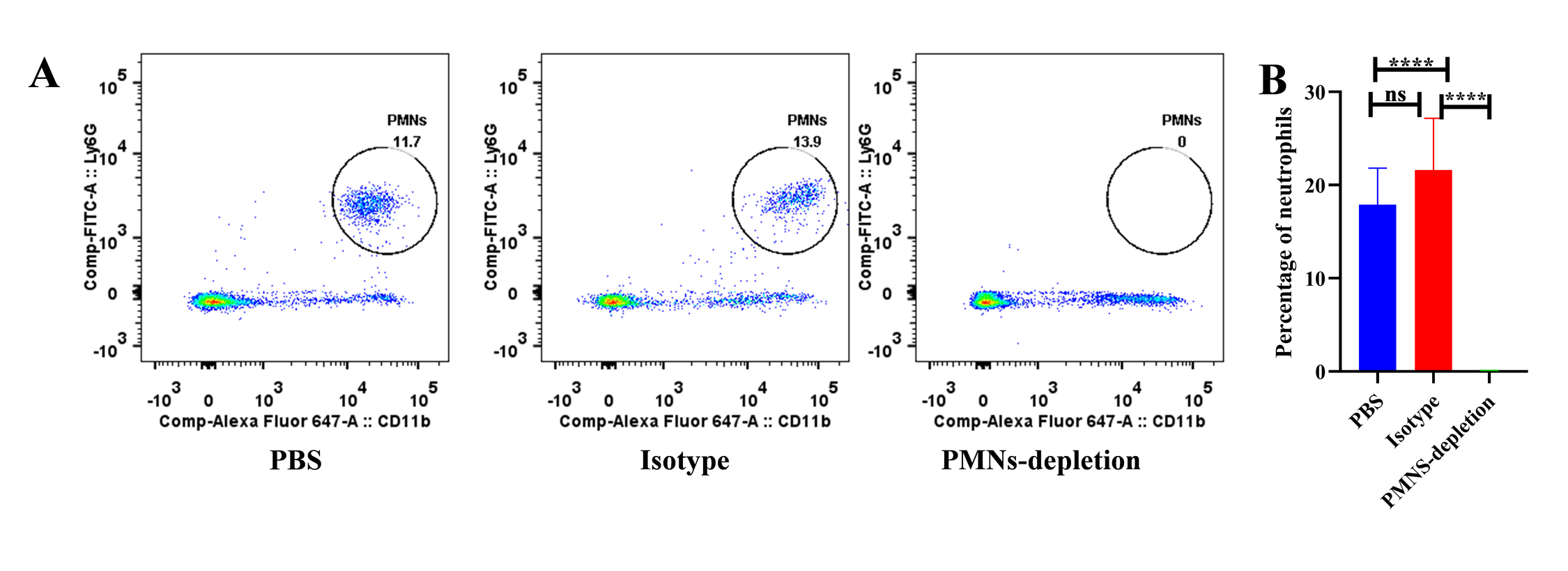

Supplement: S2 Fig — (A) Flow chart of representative neutrophils (CD11b+Ly6G+) as a percentage of leukocytes (CD45+) in mice receiving PBS, isotype control and 1A8 antibody (PMNs-depletion) for 3 days. (B) Statistical histogram of neutrophil percentages in peripheral blood of mice under different treatments. Statistical significance was determined by one-way ANOVA test (*, P<0.05; **, P<0.01; ***, P<0.0001, ****, P<0.0001.) (TIF) [file pntd.0012986.s002.tif]

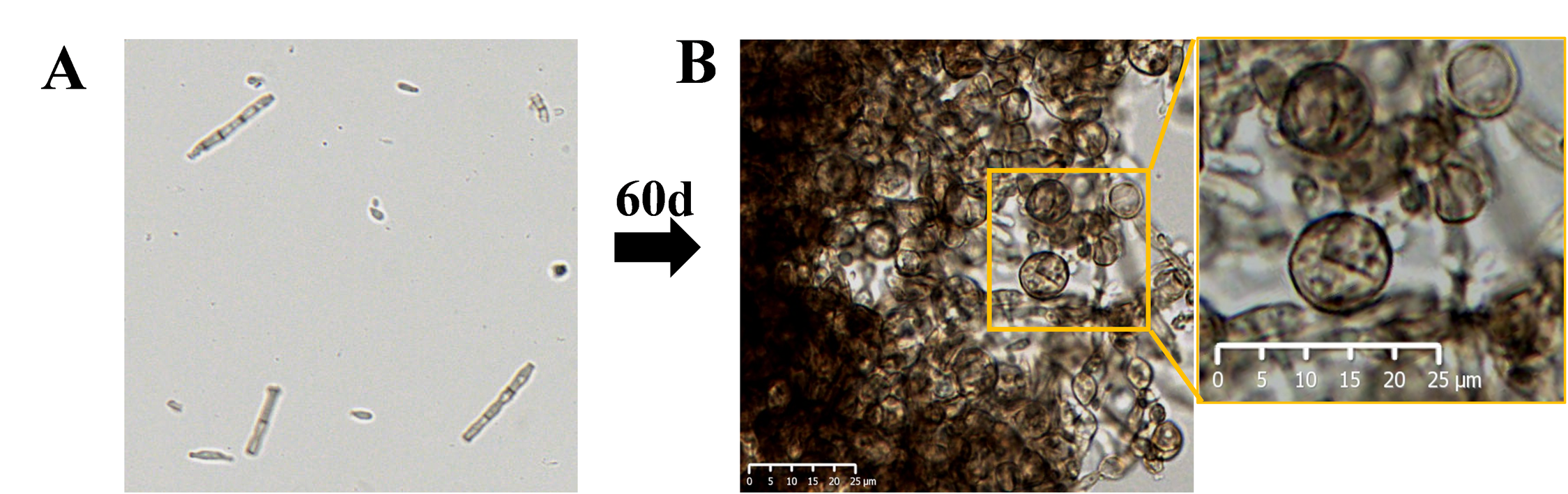

Supplement: S3 Fig — F. monophora conidia or short hyphae were successfully transformed into sclerotic cells after 60 days induction in vitro. (TIF) [file pntd.0012986.s003.tif]
